# MAT2A inhibition suppresses inflammation in *Porphyromonas gingivalis*-infected human gingival fibroblasts

**DOI:** 10.1080/20002297.2023.2292375

**Published:** 2023-12-15

**Authors:** Lishan Jiang, Jingwen Li, Kun Ji, Lang Lei, Houxuan Li

**Affiliations:** a Nanjing Stomatological Hospital, Affiliated Hospital of medical School, Nanjing University, Nanjing, China; b Central Laboratory of Stomatology, Nanjing Stomatological Hospital, Affiliated Hospital of medical School, Nanjing University, Nanjing, China

**Keywords:** S-adenosylmethionine, MAT2A, inflammation, gingival fibroblasts, Porphyromonas gingivalis

## Abstract

**Background:**

Methionine adenosyl transferase II alpha (MAT2A) is the key enzyme to transform methionine into S-adenosylmethionine (SAM), the main methylgroup donor involved in the methylation. The purpose of our study wasto explore whether MAT2A-mediated methionine metabolism affected theexpression of inflammatory cytokines in human gingival fibroblasts(hGFs).

**Methods:**

Both healthy and inflamed human gingiva were collected. HGFs werecultured and treated with P. gingivalis, with or without MAT2Ainhibitor (PF9366), small interference RNA (siRNA), or extrinsic SAMpretreatment. The levels of inflammatory cytokines were detected byreal-time PCR, western blotting, and ELISA. SAM levels were detectedby ELISA. The nuclear factor-kappa B (NF-κB) and mitogen-activatedprotein kinase (MAPK) pathway was explored by western blotting.

**Results:**

The expression of MAT2A was increased in the inflamed tissues. P.gingivalis infection promoted the expression of MAT2A and SAM inhGFs. Meanwhile, PF9366 and MAT2A-knockdown significantly decreasedexpression of inflammatory cytokines and SAM production. PF9366inhibited activation of NF-κB/MAPK pathway in P. gingivalis-treatedhGFs.

**Conclusions:**

MAT2A-mediated methionine metabolism promoted P. gingivalis-inducedinflammation in hGFs. Targeting MAT2A may provide a novel therapeuticmethod for modulating periodontitis.

## Introduction

Periodontal diseases impact the integrity of periodontal tissues, including the gingival tissue, periodontal ligament, and alveolar bone [1,2]. They are regarded as a disruption of host-microbiome homeostasis and the development of a dysbiosis [3]. This process is driven by the presence of risk factors and/or key pathogens, characterized by an increase in biomass and altered prevalence of various taxa. The loss of homeostasis between the resident microbiome and the host defense system may lead to the excessive proliferation of periodontal pathogens [4], such as *Porphyromonas gingivalis* (*P. gingivalis*) [5], *Tannerella forsythia (T. forsythia)* [6,7], and *Prevotella intermedia (P. intermedia)* [8]. *P. gingivalis* produces gingipain, one of the cysteine endo-proteases [9,10], and lipopolysaccharide (LPS) [11] to subvert the host immune system and thus promote a dysbiotic state. Following the invasion of anaerobic microorganisms into the gingival tissues, the host may release inflammatory signals to recruit immune cells to clear the pathogenic factors, and such process may also lead to the exacerbation of periodontal destruction [3].

Gingival fibroblasts comprise the most abundant resident cells in gingival connective tissues. By expressing an array of pattern recognition receptors on the cell membrane, they can sense the invasion of periodontal pathogens, producing inflammatory cytokines to trigger inflammatory responses and exacerbate inflammation [12], such as interleukin (IL)-1β, tumor necrosis factor-alpha (TNF-α), IL-6, and monocyte chemoattractant protein-1 (MCP-1) [13]. Furthermore, inflammatory modulators released by gingival fibroblasts can activate the formation of osteoclasts and induce alveolar bone loss [14]. The inflammatory responses to microbial infection are accompanied by vast changes in the glutathione, purine, and pyrimidine metabolism in gingival fibroblasts [15]. Pathogen infection also promotes changes in the amino acid metabolism in immune cells, such as macrophages, neutrophils, and T lymphocytes [16,17]; however, whether amino acid metabolism participated in the development of periodontitis has seldom been studied.

Methionine is one of the necessary amino acids in organisms, which is an important substrate involved in one-carbon metabolism. Methionine is converted by methionine adenosyltransferase (MAT1A and MAT2A) into S-adenosylmethionine (SAM) [18], which is metabolized by various N-methyltransferases (MTs) into S-adenosylhomocysteine (SAH), while the S-adenosylhomocysteine hydrolase (AHCY) converts SAH to homocysteine. Importantly, SAM is also a precursor to spermine synthesis. SAM is first switched to S-adenosylmethioninamine (also named as decarboxylated S-adenosylmethionine [dcSAM]) by the enzyme adenosylmethionine decarboxylase 1 (AMD1), and dcSAM is required to convert spermidine into spermine by spermine synthase (SMS) [19]. SAM is the active form of methionine present intracellularly, which acts as a general methyl-group donor in cells [20]. Methylated modification, such as DNA methylation, N6-methyladenosine and histone methylation, is one of the most common epigenetic modifications and has been proved to be very important for many biological processes, especially inflammation [21].

MAT is the key enzyme of one-carbon metabolism. Its family is encoded by three genes: MAT1A, MAT2A, and MAT2B. Most dietary methionine is processed in the liver by hepatic MAT1A. Extrahepatic SAM is synthesized by the enzyme MAT2A, and its activity is regulated by its binding to MAT2B [22]. Most of the research on the involvement of MAT family proteins in the occurrence and development of diseases mainly focuses on cancer, especially liver cancer [23]. MAT2A and MAT2B are also involved in the occurrence and development of colorectal cancer [24], gastric cancer [25], cervical cancer [26], mixed-lineage leukemia [27], colitis [28], and osteoclastogenesis [29].

Metabolic rewiring is important in supporting the combat against the invasion of microorganisms [17,30]. However, whether the MAT2A-mediated methionine metabolism promoted inflammatory cytokine production and activation of the pro-inflammatory signaling pathway has never been studied so far. Thereby, the main purpose of this study was to explore the potential role of MAT2A-mediated methionine metabolism in inflammation in *P. gingivalis*-stimulated hGFs and further investigate the possible mechanism underlying the interaction between MAT2A and inflammation.

## Materials and methods

### Collection of gingival tissue from the subjects

Gingival tissue specimens, including the marginal gingival epithelium and connective tissues, were collected from patients of the Nanjing Stomatological hospital. This study was approved by the Medical Ethics Committee of Nanjing Stomatological Hospital, Medical School of Nanjing University. The ethical approval number is NJSH-2022NL-35. All subjects gave written informed consent following the Declaration of Helsinki of 2013. Healthy periodontal tissues (from 3 men and 3 women, aged 36.67 ± 8.69 years) were collected during clinical crown lengthening, open flap curettage, and wisdom tooth extraction. While periodontitis tissues (from 6 men and 4 women, aged 42.60 ± 8.33 years) were taken during the extraction of hopeless teeth with three degrees of mobility from patients with stage III/IV grade B periodontitis, the sampling teeth displayed increased probing depths (>6 mm) and positive BOP. All patients met the following inclusion criteria: no consumption of antibiotics or anti-inflammatory medications within the last 3 months, no previous periodontal treatment, and the maintenance of systemic health. The exclusion criteria included any systemic diseases that might affect periodontal disease, immunodeficiency, and previous smoking history or current smoking.

### Cell culture

Primary human gingival fibroblasts (hGFs) were established from gingival tissue samples from healthy individuals (3 donors: 2 men, aged 23 and 15 years, and 1 woman, aged 18 years). An explant method was utilized to collect and culture gingival fibroblasts as described previously [31]. Briefly, the pieces sliced from healthy gingival connective tissues were cultured in Dulbecco’s modified Eagle medium (DMEM; Gibco, USA), 20% fetal bovine serum (FBS; Gibco, USA), penicillin (100 U/mL; Hyclone, USA), and streptomycin (100 mg/mL; Hyclone, USA). The culture conditions were maintained at 37°C, 5% CO_2_, and 95% air. The isolated hGFs were sub-cultured in DMEM, 10% FBS, penicillin (100 U/mL), and streptomycin (100 mg/mL). Cells in the 3rd to 6th generations were used for the experiment.

### Bacterial culture and infection of human gingival fibroblasts

*P. gingivalis* (ATCC 33,277) was cultured in the brain heart infusion (BHI; Oxiod, USA) medium supplemented with hemin (5 mg/L; Alfa Aesar, USA) and menadione (1 mg/L; Alfa Aesar, USA) in the anaerobic condition (85% N_2_, 5% H_2_, and 10% CO_2_). The bacteria were obtained by centrifugation, washed by PBS twice, and re-suspended in DMEM. The concentration of bacteria was determined by the spectrophotometer at 600 nm with an OD value of 1 equal to 1 × 10^9 CFU/mL. The hGFs were infected with *P. gingivalis* at the exponential proliferation stage. The cells were seeded on six-well plates overnight, pretreated with the MAT2A inhibitor PF9366 (S0435; Selleck Chemicals, USA), SAM (S5109; Selleck Chemicals, USA), or small interference RNA transfection if necessary, followed by incubation with antibiotic-free DMEM and 10% FBS, and at a density of 2 × 10^5^ cells/2 mL per well before infection.

### Immunohistochemistry (IHC) staining

Sixteen gingival tissues (6 health and 10 periodontitis) were treated according to previous reports [32]. The tissues were collected and fixed in 4% paraformaldehyde. Several paraffin sections were prepared (4 μm). The sections were incubated with anti-MAT2A (1:200; 55309–1-AP; Proteintech, China) overnight at 4°C. Then, the slides were washed three times with phosphate-buffered saline and incubated with goat anti-rabbit IgG (MaxVision, China) at room temperature for 30 min. Diaminobenzidine (DAKO, USA) was used as a chromogenic agent to detect antibody binding. Six fields of vision were randomly selected from each slice. IHC profiler was used to count the positive cells automatically. The product of the percentage of positive cells and the staining intensity of positive cells were used to score.

### MAT2A-knockdown

We conduct the transfection of small interference RNA (siRNA) with Lipofectamine 2000 (Thermo Fisher Scientific, USA) in Opti-MEM medium (Thermo Fisher Scientific, USA) at 100 nM based on the manufacturer’s protocols. A local company (Public Protein/Plasmid Library, China) designed and synthesized the MAT2A and scramble siRNA constructs. Sequences of MAT2A siRNA were as follows (a) 5’-AGAUAAGAUUUGUGACCAATT-3’ and 3’-UUGGUCACAAAUCUUAUCUTT-5’, (b) 5’-CGAAAUACCUUGAUGAGGATT-3’ and 3’-UCCUCAUCAAGGUAUUUCGTT-5’ (c) 5’-GAGCAACAGUCACCAGAUATT-3’ and 3’-UAUCUGGUGACUGUUGCUCTT-5’. The sequence of the scramble control siRNA was designed as 5’-UUCUCCGAACGUGUCACGUTT-3’ and 3’-ACGUGACACGUUCGGAGAATT-5’. HGFs were plated on six-well plates, incubated with siRNAs for 72 h and at a density of 2 × 10^5^ cells/2 mL per well before further treatment. Subsequently, the cells were harvested to determine the knockdown effect by qPCR and western blotting.

### Real-time PCR

Total RNA was extracted from hGFs using an RNA Plus kit (Tiangen, China) and the purity of it was tested via a Nanodrop (Thermo Fisher Scientific, USA) at 260 nm. The RNA was reverse transcribed into cDNA immediately using a reverse transcription kit (Vazyme, China) according to the manufacturer’s instructions. The primers used for qPCR were synthesized and provided by GenScript and the sequences are shown in Table 1. The efficiency of PCR was checked via PrimerBank. Real-time PCR was conducted in triplicate using SYBR Green Master MIX (Vazyme, China) in a Viia 7 Real-Time PCR System (ABI, USA). The mixture was heated at 95°C for 10 min, followed by 40 cycles of denaturation at 95°C for 15 s and a combined annealing/extension step at 60°C for 1 min. Relative quantification was calculated by the comparative 2^–ΔΔCt^ method after normalized to β-actin transcription [33].

**Table 1.** Primer sequences used for real-time PCR.

Gene	Sequence (5’ to 3’)	GeneBank accession
MAT2A	Forward ACCAGATATTGCTCAAGGTGTTC Reverse GCATAGCCAAACATTAAGCCCT	NM_005911
AMD1	Forward AGTCGGGTAATCAGTCAGCCA Reverse ACTCTCACGAGTGACATCCTTT	NM_001634
SMS	Forward TAGTGGGGATGTTAATTTGGCAG Reverse CCACACGTTTTTCGCATGTATTT	NM_004595
AHCY	Forward TAGCAGGCTATGGTGATGTGG Reverse ATGGGGTCAATCTCGGTGATG	NM_000687
IL-1β	Forward TTCCTGTTGTCTACACCAATGC Reverse CGGGCTTTAAGTGAGTAGGAGA	NM_000576
TNF-α	Forward GGAGGGGTCTTCCAGCTGGAGA Reverse CAATGATCCCAAAGTAGACCTGC	NM_000594
IL-6	Forward GAAAGCAGCAAAGAGGCACT Reverse TTTCACCAGGCAAGTCTCCT	NM_000600
MCP-1	Forward CAGCCAGATGCAATCAATGCC Reverse TGGAATCCTGAACCCACTTCT	NM_002982
Actin	Forward GTGGGGCGCCCCAGGCACCA Reverse CGGTTGGCCTTGGGGTTCAGGGGGG	NM_001101

### Western blotting

Total proteins from cultured hGFs were extracted using radioimmunoprecipitation assay (RIPA) lysis buffer on the ice. The protein concentration was determined via a Nanodrop. Proteins at 10 μL per lane were separated by 4–20% SDS-PAGE (Beyotime, China) and then transferred onto PVDF membranes (Merck, Germany), which were blocked with 5% bovine albumin and then incubated with primary antibodies to MAT2A (1:1000; 55309–1-AP; Proteintech, China), IL-1β (1:1000; BJ11258993; Bioss, China), TNF-α (1:400; GB11188; Servicebio, China), IL-6 (1:1000; 21865–1-AP; Proteintech, China), MCP-1 (1:1500; ab25124; Abcam, USA), P65 (1:1000; 8242S; CST, Germany), Erk (1:1500; GB11560; Servicebio, China), P38 (1:1000;8690S; CST, Germany), JNK (1:1000; AF6318; Affinity, USA), p-P65 (1:1000; 3033S; CST, Germany), p-Erk (1:1000; AF1015; Affinity, USA), p-P38 (1:1000; 4511S; CST, Germany), p-JNK (1:1000;4668S; CST, Germany) and β-ACTIN (1:50000; 66009-l-Ig; Proteintech, China). After the incubation step, membranes were washed again 3 times and incubated with secondary antibodies. Images were provided with ImageQuant LAS 4000 (USA). The intact optical density for the protein band was calculated by the ImageJ software.

### Enzyme-linked immunosorbent assay (ELISA)

Protein levels of IL-6 (Human IL-6 ELISA Kit, LA137702H, Nanjing Lapuda, China), MCP-1 (Human MCP-1 ELISA Kit, LA137709H, Nanjing Lapuda, China), and SAM (Human SAM ELISA Kit, CEG414Ge02, China) in hGFs culture supernatants were measured after 24 h of stimulation. Samples were concentrated or diluted to the appropriate concentration. Protocols were followed according to the manufacturer’s instructions for each specific kit. ELISA were conducted in triplicate, and the optical density of each well was determined immediately by a microplate reader at 450 nm.

### Cytotoxicity of PF9366 in hGfs

The viability of hGFs treated with PF9366, a MAT2A inhibitor, (S0435; Selleck Chemicals, USA) was quantified using Cell Counting Kit-8 (CCK-8, DOJINDO, Japan). Briefly, hGFs were seeded on 96-well plates at 5000 cells per well and incubated overnight. PF9366 was then added to the wells at final concentrations of 0, 1, 10, and 50 μM. After incubation for 24 h, 10% CCK-8 solution in culture medium was added to each well. The optical density at 450 nm was determined using a microplate reader after incubation for 2 h.

### Statistical analysis

All data were shown as mean ± standard deviation of three independent experiments. Significant differences between two groups were determined using Student’s *t*-test, while multiple comparisons among four groups were performed by one-way analysis of variance with Dunnett’s test. Statistical values were revealed in the figures performed by GraphPad Prism.

## Results

### MAT2A expression was upregulated in inflamed gingival tissues

**Figure 1.**
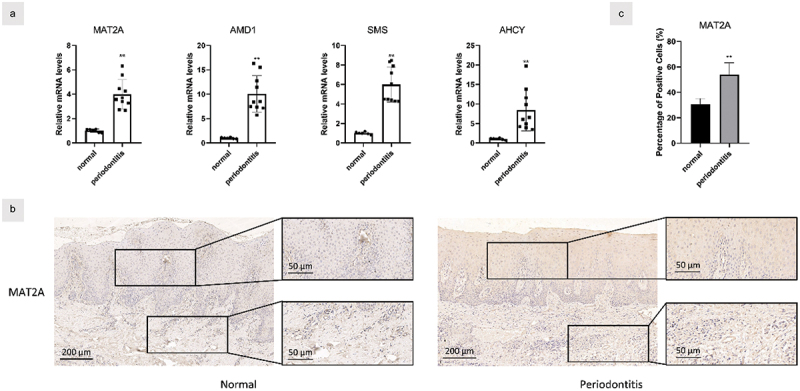
MAT2A expression was upregulated in inflamed gingival tissues.

First, we explored the expression of methionine metabolism-related genes and inflammation-associated genes in the gingival tissues. Elevated mRNA expression of key enzymes of methionine metabolism, such as MAT2A, AMD1, SMS, and AHCY, were observed in the inflamed gingival tissues (Figure 1a).

(a) Gene transcription in the healthy (*n* = 6) and inflamed (*n* = 10) gingiva was measured by real time qPCR. (b&c) Samples from healthy (*n* = 6) and inflamed gingiva (*n* = 10) were stained by the immunolohistological chemistry. Data are presented as the mean ± SD. **p < 0.01 versus control.

In addition, we examined the protein expression of MAT2A in the healthy and inflamed tissues by immunohistochemistry (Figures 1b,c). More positive staining of MAT2A could be detected in the inflamed gingival tissues than in the healthy ones. These results showed an upregulation of MAT2A in the local periodontal microenvironment of periodontitis patients, and MAT2A acts as a pivotal regulator in the methionine metabolism, suggesting that the methionine metabolism is enhanced in periodontal tissue.

### P. gingivalis enhanced methionine metabolism in hGFs

**Figure 2.**
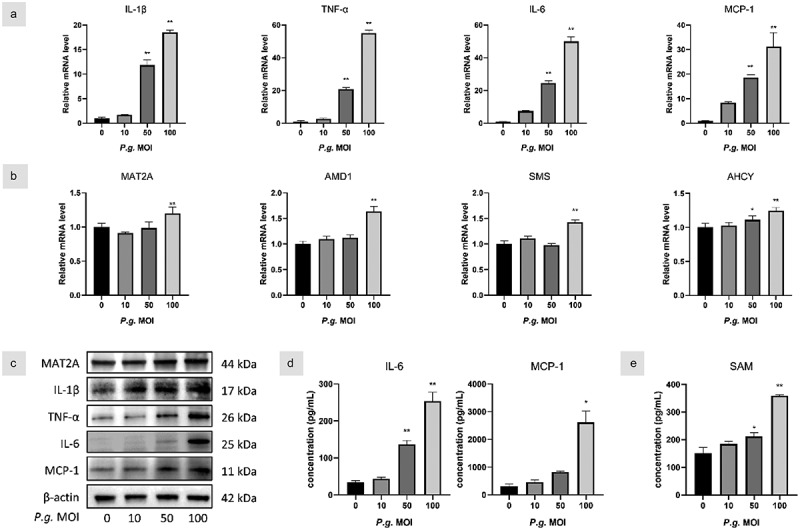
*P. gingivalis* enhanced methionine metabolism in hGFs.

To further investigate the role of methionine metabolism in periodontitis, hGFs were treated with *P. gingivalis* in a concentration-dependent manner. Levels of IL-1β, TNF-α, IL-6, and MCP-1 mRNA were significantly upregulated by *P. gingivalis* stimulation in hGFs (Figure 2a). Meanwhile, the infection increased the mRNA transcription of MAT2A, AMD1, SMS, and AHCY mRNA with the same MOI (Figure 2b). Western blot analysis showed the same trend under the treatment for 24 h (Figure 2c). Furthermore, inflammatory cytokines and SAM levels were remarkably increased by *P. gingivalis* treatment compared with uninfected hGFs (Figures 2d,e). These results indicated that the inflammation model was successfully established in vitro and *P. gingivalis* treatment promoted MAT2A-mediated methionine metabolism in hGFs.

(a&b) hGFs were infected with *P. gingivalis* (MOI = 10, 50, and 100) for 3 h, and gene transcription levels were assessed by real-time qPCR. (c) Protein expression was analyzed by Western blot at 24 h. (d) IL-6, MCP-1, and (e) SAM levels in hGFs were measured using ELISA. Data are presented as the mean ± SD (*n* = 3). *p < 0.05; **p < 0.01 versus control.

### MAT2A inhibition decreased P. gingivalis-induced inflammatory response in hGFs

**Figure 3.**
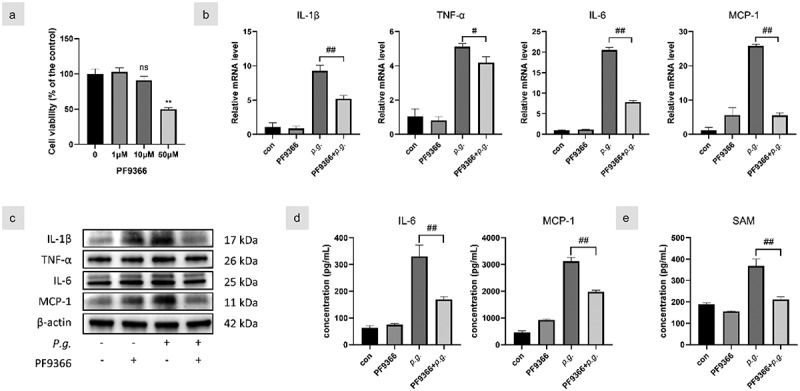
MAT2A inhibition decreased *P. gingivalis*-induced inflammatory responses in hGfs.

Since *P. gingivalis* infection activates methionine metabolism in hGFs, we next examined if methionine metabolism affects the production of inflammatory cytokines in response to *P. gingivalis* challenge. We utilized PF9366, a specific inhibitor of MAT2A, to pretreat *P. gingivalis*-infected cells. The viability of hGFs treated with PF9366 was quantified using the Cell Counting Kit-8 and was not significantly changed at 10 μM (Figure 3a). PF9366 significantly attenuated transcript and protein expression of inflammatory cytokines (Figures 3b–d). In addition, SAM levels were decreased by PF9366 (Figure 3e).

(a) The viability of hGFs treated with PF9366 was quantified using Cell Counting Kit-8 and hGFs were pretreated with PF9366 (10 μM) and then stimulated with *P. gingivalis* (MOI = 100) in subsequent experiments. (b) Gene transcription of inflammatory cytokines was assessed by qPCR at 3 h and (c) protein levels were detected by Western blot at 24 h. (d) IL-6, MCP-1, and (e) SAM in the culture supernatants of 24 h were detected by ELISA. Data are presented as the mean ± SD (*n* = 3). *p < 0.05; **p < 0.01 versus control. #*p* < 0.05; ##*p* < 0.01.

### MAT2A-knockdown reduced inflammation in P. gingivalis-infected hGFs

**Figure 4.**
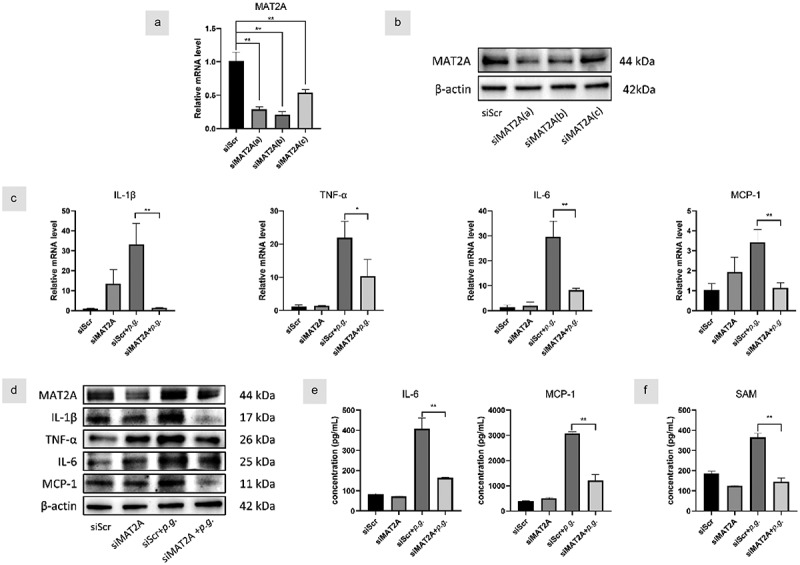
MAT2A-knockdown reduced inflammation in *P. gingivalis*-infected hGFs.

To exclude the possible unspecific effects of the chemical inhibitor, we next utilized specific mat2a siRNA to verify the effects of MAT2A on the production of inflammatory cytokines. We explored the efficacy of several siRNA plasmids to knock down MAT2A. MAT2A siRNA(b) drastically reduced the mRNA transcription and protein level of MAT2A (Figures 4a,b). Similarly, MAT2A knockdown notably lessened inflammatory cytokines compared with the *P. gingivalis* group (Figures 4c–e). As expected, SAM level was also diminished after MAT2A knockdown in hGFs (Figure 4f). Therefore, MAT2A-knockdown suppressed inflammation in *P. gingivalis*-treated hGFs.

HGFs were transfected with siRNA for 72 h. (a&b) The efficiency of three siRNA plasmids to knock down MAT2A was detected by Western blot and qPCR, respectively. HGFs with MAT2A or scrambled siRNA were infected with *P. gingivalis* (MOI = 100). (c) Gene transcription of inflammatory cytokines was assessed by qPCR at 3 h and (d)protein levels were detected by Western blot at 24 h. (e) IL-6, MCP-1, and (f) SAM in the culture supernatants of 24 h were detected by ELISA. Data are presented as the mean ± SD (*n* = 3). *p < 0.05; **p < 0.01.

### SAM synergistically promotes P. gingivalis-induced inflammation in hGFs

**Figure 5.**
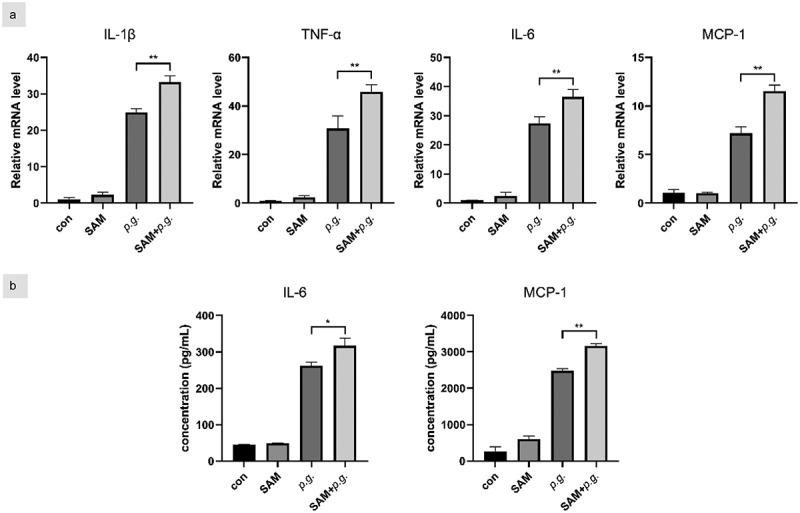
SAM synergistically promotes *P. gingivalis*-induced inflammation in hGFs.

SAM, the MAT2A metabolite, is a main methyl donor in methylated modification. To further confirm the effect of MAT2A on the inflammatory reaction in hGFs, we supplied extrinsic SAM (10 μM) to pre-culture *P. gingivalis*-infected cells. Compared to the *p.g*. group, increased mRNA levels of all inflammatory cytokines were observed for SAM+*p.g*.-treated cells (Figure 5a). Significant higher protein levels of IL-6 and MCP-1 were also found in the SAM+*p.g*. group (Figure 5b). No significant difference of the inflammatory cytokines between the control and SAM group was detected, as analyzed by qPCR and ELISA. Thus, SAM has a positive synergistic effect on the inflammatory response to *P. gingivalis* infection.

HGFs were pre-cultured with medium in the presence of SAM (10 μM) for 2 days and then infected with *P. gingivalis* (MOI = 100) for 3 h for qPCR and 24 h for ELISA. (a) Gene transcription of inflammatory cytokines was assessed by qPCR. (b) IL-6 and MCP-1 in the culture supernatants were detected by ELISA. Data are presented as the mean ± SD (*n* = 3). *p < 0.05; **p < 0.01.

### NF-κB/MAPK signaling pathway was involved in the enhanced MAT2A expression

**Figure 6.**
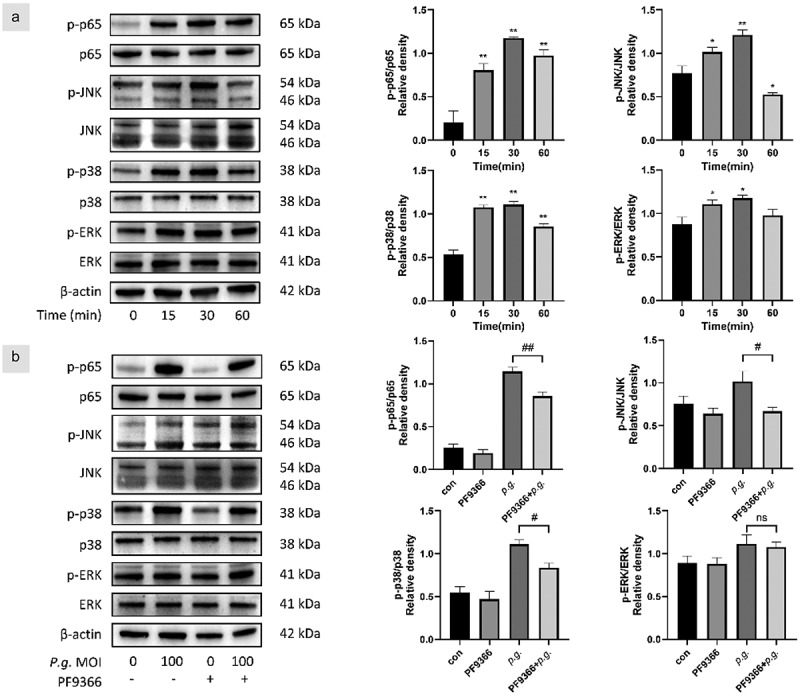
NF-κB/MAPK pathway was involved in the enhanced MAT2A expression.

Since we have demonstrated that MAT2A restrains *P. gingivalis*–mediated inflammatory responses, we next sought to delineate how MAT2A affects inflammatory responses in hGFs upon the challenge of *P. gingivalis*. NF-κB, one of the key regulators of metabolic factors, plays a pivotal role in multiple steps of inflammation. As shown in Figure 6a, *P. gingivalis* stimulated the activation of NF-κB and MAPKs, indicating *P. gingivalis* treatment activated NF-κB/MAPK pathway. Furthermore, we explored whether the NF-κB/MAPK pathway activation was involved in MAT2A and inflammatory cytokines upregulation in hGFs. The impact of PF9366 on this pathway was examined by western blotting represented in Figure 6b. PF9366 suppressed the NF-κB/MAPK pathway, revealed by decreased phosphorylation levels of p65, p38, and JNK. Meanwhile, the p-ERK pathway was slightly affected. Hence, MAT2A and proinflammatory cytokines expression were related to NF-κB/MAPK pathway in hGFs.

(a) HGFs were time-dependently infected with *P. gingivalis* (MOI = 100). The total and phosphorylated protein expression levels of NF-κB and MAPK were analyzed by Western blot. (b) HGFs were pretreated with or without PF9366 for 0.5 h and then treated with *P. gingivalis* for 0.5 h. The total and phosphorylated protein expression levels of NF-κB and MAPK were analyzed. Data are presented as the mean ± SD (*n* = 3). *p < 0.05; **p < 0.01 versus control. #*p* < 0.05; ##*p* < 0.01.

## Discussion

Amino acids are key elements in the inflammatory responses to microbial infection in immune cells for building proteins and nucleotides [34]; in addition, they are also critical for generating metabolic intermediates, which participate in a variety of physiological processes [35]. Besides its contribution to sulfur metabolism, methionine donates methyl groups for methylation of proteins and nucleotides to modulate their function, facilitating cytokine gene expression, and both innate and adaptive immune memory [36]. In the present study, we for the first time reported that methionine metabolism was elevated in the gingival tissues of periodontitis patients, and MAT2A inhibition can reduce SAM generation and inflammatory response in hGFs by inhibiting the NF-κB/MAPK signaling pathway. These results suggest that MAT2A-mediated methionine metabolism promoted inflammation in periodontitis.

MAT1A and MAT2A synthesize SAM in hepatic tissues and extrahepatic tissues, respectively. Since SAM is the principal donor of the methyl group in multiple physiological processes, MAT1A and MAT2A may participate in various inflammatory diseases, such as experimental autoimmune encephalomyelitis [37], alcoholic hepatitis, non-alcoholic fatty liver diseases [38], and inflammatory bowel diseases [28]. Previously, it has been demonstrated that MAT2A promoted SAM generation and enhanced IL-1β transcription in LPS-stimulated macrophages [39]. The elevated MAT2A expression in both the inflamed gingival tissues and *P. gingivalis*-infected GFs indicated that MAT2A-mediated methionine metabolism may participate in the development of periodontitis.

SAM is not only involved in the de novo synthesis of cellular glutathione (GSH) but also acts as a major unique methyl donor in DNA and histone methylation. Like elevated SAM levels in LPS-treated macrophages [39], we observed increased SAM generation in GFs following *P. gingivalis* infection. Pfalzer et al. reported that SAM reduced proinflammatory TNF-α and promoted anti-inflammatory IL-10 in LPS-stimulated macrophages [40], whereas Yu et al. found out that SAM generation promoted IL-1β production in macrophages. Therefore, SAM production may play a different role in regulating the process of inflammation. Our present study showed that *P. gingivalis* infection promoted S-Adenosyl-L-methionine (SAM) production in GFs.

To explore how MAT2A-mediated SAM production regulates pro-inflammatory cytokine production, we focused on the inflammatory NF-κB/MAPK signaling pathway, which is critical in the production of cytokines, chemokines, and adhesion molecules [41]. In our study, *P. gingivalis* triggered the activation of NF-κB/MAPK signaling pathway, and suppression of MAT2A inhibited expression of both NF-κB/MAPK pathway and inflammation. This pathway involves activation and nuclear translocation of NF-κB and many compounds can reduce inflammatory cytokines in hGFs by blocking NF-κB activation [42–44]. Moreover, the promoter activity of MAT2A was associated with NF-κB and AP-1 elements [45] and the inhibition of MAT2A significantly suppressed NF-κB/MAPK pathway [29], which concurred with our findings.

SAM acts as a general methyl-group donor in cells, and methylated modification, like DNA methylation and histone methylation, is highly likely to participate in the process of periodontitis. The most studied form of DNA methylation is the covalent addition of methyl groups to the fifth carbon on the cytosine base (5 mC) within the CpG islands of the promoter region of a gene, catalyzed by DNA methyltransferases (DNMTs) [46]. DNA methylation is generally regarded as a stable inhibitory regulator of gene expression [47]. A hypomethylation of signal transducers and activators of transcription 5 (STAT5) promoter and prostaglandin-endoperoxide synthase 2 (PTGS2) promoter, increased methylation at two CpG sites of TNF-α and TLR2 gene in gingival tissues from chronic periodontitis patients has been reported [48]. Further studies are needed to explore how MAT2A-mediated methionine metabolism and SAM production affect DNA methylation and its interaction with individual DNMTs.

In addition to DNA methylation, MAT2A-mediated SAM production may also affect the histone methylation status. Histone methylation is involved in both activation and repression of gene expression. The trimethylation of histone H3 at lysine-4, −36, and −79 (H3K4me3, H3K36me3, and H3K79me3) results in active marks, whereas trimethylation of H3 at lysine-9 and −27 (H3K9me3 and H3K27me3) indicates repressive marks [49]. In response to LPS stimulation, H3 lysine 4 trimethylation (H3K4me3) enrichment was increased on the promoters of inflammatory response genes, such as C-C Motif chemokine ligand 5(CCL5) and IL-1β in the periodontal ligament stem cells [50], while LPS treatment and SAM generation promoted H3 lysine 36 trimethylation(H3K36me3) for IL-1b production in bone marrow-derived macrophages(BMDMs) [39]. Further studies are needed to explore how *P. gingivalis* infection and MAT2A upregulation imprint the histone methylation status in the inflamed gingival tissues and GFs.

There are also some limitations to this study. In this study, our results have shown a discrepancy in MAT2A production between in vitro assays and clinical samples. The difference between the samples we used could be a major reason for this discrepancy. Unlike an in vitro monoculture, gingival tissue includes not only fibroblasts but also monocytes, macrophages, neutrophils, and epithelial cells. Further studies are needed to examine the function of MAT2A in immune cells following *P. gingivalis* infection. In addition, more research is needed to explore how the various pathogenic factors, such as gingipains and LPS, alter MAT2A-mediated methionine metabolism and SAM production. Moreover, animal studies are needed to study whether MAT2A inhibition can reduce the inflammation in the periodontal tissues.

## Conclusion

MAT2A-mediated methionine metabolism participated in the inflammatory cytokines production in hGFs following *P. gingivalis* infection and MAT2A activation promoted NF-κB/MAPK signaling pathway. Pharmaceutical inhibition of MAT2A may be a potential target to modulate periodontal inflammation.
